# Association of Atherogenic Indices With Disease Activity in Rheumatoid Arthritis Patients: A Cross-Sectional Study

**DOI:** 10.7759/cureus.112234

**Published:** 2026-07-07

**Authors:** Rajeev Roy, Harsh Anadkat, Tazean Zahoor Malik, Pooja Parmar, Sajidali S Saiyad, Grishma H Chavda, Gnanadesigan Ekambaram, P Padmavathi, Prasanta Chatterjee Biswas

**Affiliations:** 1 Preventive Medicine, Panimalar Medical College Hospital and Research Institute, Chennai, IND; 2 Department of Medicine, Sterling Ramakrishna Speciality Hospital, Gandhidham, IND; 3 Community Medicine, Sher-i-Kashmir Institute of Medical Sciences (SKIMS) Soura, Srinagar, IND; 4 Department of Anatomy, J and D Institute of Nursing, Surat, IND; 5 Department of Physiology, Pacific Medical College and Hospital, Pacific Medical University, Udaipur, IND; 6 Obstetrics and Gynaecology, Dr. N. D. Desai Faculty of Medical Science and Research, Nadiad, IND; 7 Physiology, Nootan Medical College and Research Centre, Sankalchand Patel University, Visnagar, IND; 8 Biochemistry, Nootan Medical College and Research Centre, Sankalchand Patel University, Visnagar, IND; 9 Department of Biostatistics, Parul University, Vadodara, IND

**Keywords:** atherogenic index of plasma, atherogenic indices, biomarkers, cardiometabolic risk, cardiovascular risk, das28-crp, disease activity, dyslipidemia, inflammation, rheumatoid arthritis

## Abstract

Background: Rheumatoid arthritis (RA) is a chronic autoimmune inflammatory disorder associated with accelerated atherosclerosis and increased cardiovascular morbidity. Atherogenic indices derived from routine lipid parameters have emerged as potential markers of cardiovascular risk; however, their relationship with disease activity in RA remains incompletely understood, particularly in Indian populations.

Objective: To evaluate the association between atherogenic indices and disease activity in patients with rheumatoid arthritis and to assess their potential utility as biomarkers of inflammatory burden and cardiometabolic risk.

Methodology: This cross-sectional observational study included 100 patients with rheumatoid arthritis (RA) who met the 2010 American College of Rheumatology/European League Against Rheumatism (ACR/EULAR) classification criteria and 50 years of age- and sex-frequency-matched control participants without rheumatoid arthritis or other inflammatory rheumatic diseases. Clinical, anthropometric, laboratory, and lipid profile data were collected. Disease activity was assessed using the Disease Activity Score in 28 joints with C-reactive protein (DAS28-CRP). Atherogenic indices, including Castelli's Risk Index-I (CRI-I), Castelli's Risk Index-II (CRI-II), Atherogenic Index of Plasma (AIP), Atherogenic Coefficient (AC), and non-high-density lipoprotein cholesterol (non-HDL-C), were calculated and analyzed in relation to disease activity measures.

Results: Patients with RA exhibited significantly higher triglycerides, low-density lipoprotein cholesterol (LDL-C), very low-density lipoprotein cholesterol (VLDL-C), non-high-density lipoprotein cholesterol (non-HDL-C), Castelli's Risk Index-I (CRI-I), Castelli's Risk Index-II (CRI-II), Atherogenic Index of Plasma (AIP), and Atherogenic Coefficient (AC), together with lower HDL-C levels compared with healthy controls (all p<0.05). Atherogenic indices increased progressively across disease activity categories, with the highest values observed among patients with high disease activity (all p≤0.001). Among the evaluated indices, AIP demonstrated the strongest correlation with Disease Activity Score in 28 joints with C-reactive protein (DAS28-CRP) (r=0.45, p<0.001), followed by CRI-II (r=0.38, p<0.001). In multivariable logistic regression analysis, AIP remained independently associated with high disease activity (odds ratio (OR)=3.12, 95% CI: 1.42-6.85, p=0.004), together with erythrocyte sedimentation rate (ESR) and BMI.

Conclusions: Atherogenic indices are significantly associated with disease activity in rheumatoid arthritis, with AIP demonstrating the strongest relationship with inflammatory burden. These findings suggest that AIP may serve as a simple, inexpensive, and readily available marker for the integrated assessment of disease activity and cardiometabolic risk in patients with RA. Prospective multicenter studies are warranted to validate its clinical utility and prognostic significance.

## Introduction

Rheumatoid arthritis (RA) is a chronic systemic autoimmune disease characterized by persistent synovial inflammation, progressive joint destruction, functional impairment, and a wide range of extra-articular manifestations. In addition to musculoskeletal disability, patients with RA experience a significantly increased risk of cardiovascular disease (CVD), which remains one of the leading causes of morbidity and premature mortality in this population. Chronic systemic inflammation, endothelial dysfunction, oxidative stress, and disturbances in lipid metabolism contribute to accelerated atherosclerosis while also influencing inflammatory disease activity, making comprehensive metabolic assessment an important component of RA management [1].

Metabolic abnormalities are increasingly recognized as important contributors to the excess cardiovascular burden observed in RA. Metabolic syndrome (MetS), characterized by central obesity, hypertension, insulin resistance, hyperglycemia, and dyslipidemia, occurs more frequently in patients with RA than in the general population. A recent systematic review and meta-analysis involving more than 13,000 patients estimated the pooled prevalence of MetS in RA to be approximately 32%, highlighting the substantial cardiometabolic burden associated with the disease [2]. Furthermore, metabolic dysfunction and chronic inflammation appear to interact bidirectionally, creating a cycle that may exacerbate inflammatory disease activity, joint damage, and cardiometabolic risk [1].

Among the metabolic alterations observed in RA, dyslipidemia plays a pivotal role in the development of atherosclerosis. Conventional lipid parameters such as total cholesterol (TC), triglycerides (TG), low-density lipoprotein cholesterol (LDL-C), and high-density lipoprotein cholesterol (HDL-C) are commonly used to assess cardiovascular risk. However, increasing evidence suggests that composite lipid-derived measures, collectively known as atherogenic indices, may provide a more comprehensive evaluation of atherogenic risk. These include the Atherogenic Index of Plasma (AIP), Castelli Risk Index-I (CRI-I), Castelli Risk Index-II (CRI-II), and Atherogenic Coefficient (AC), which reflect the balance between atherogenic and anti-atherogenic lipoproteins [3].

The potential utility of atherogenic indices in RA has attracted considerable research interest. In a hospital-based study from northeast India, Bhattacharya et al. reported significantly elevated atherogenic indices among patients with RA and demonstrated positive associations between these indices, metabolic syndrome, and disease activity measures [3]. Similarly, Dessie G observed significant relationships between atherogenic indices, C-reactive protein levels, and established cardiovascular risk factors, suggesting that these indices may reflect both inflammatory burden and disturbances in lipid metabolism in patients with RA [4]. More recently, Sreekumar et al. highlighted the high prevalence of atherothrombotic risk factors in RA and emphasized the need for improved cardiovascular risk assessment strategies in routine clinical practice [5].

Several studies have also explored the relationship between metabolic abnormalities and RA disease activity. Grzechnik and Targonska-Stepniak demonstrated that RA patients with metabolic syndrome had significantly higher disease activity scores, inflammatory markers, and ultrasound evidence of synovitis than those without metabolic syndrome, supporting a close association between metabolic dysfunction and active disease [6]. However, findings remain inconsistent across populations. Tekaya and Rouached reported a high prevalence of metabolic syndrome among RA patients but found no significant association between metabolic syndrome and disease activity indices, indicating that the relationship between cardiometabolic abnormalities and inflammatory activity may vary according to demographic and clinical characteristics [7].

Beyond RA-specific studies, recent population-based evidence has strengthened the biological link between dysregulated lipid metabolism and inflammatory joint disease. Using data from the National Health and Nutrition Examination Survey (NHANES), Ge et al. demonstrated a significant positive association between the Atherogenic Index of Plasma and arthritis prevalence, suggesting that lipid-derived atherogenic markers may have broader relevance in inflammatory musculoskeletal disorders [8]. Furthermore, recent evidence has emphasized the close relationship between chronic systemic inflammation, accelerated atherosclerosis, and cardiovascular disease in patients with rheumatoid arthritis, reinforcing the importance of comprehensive cardiovascular risk assessment in this population [9].

Despite growing evidence supporting the role of atherogenic indices in cardiovascular risk stratification among patients with RA, data regarding their relationship with inflammatory disease activity remain limited, heterogeneous, and sometimes contradictory across different populations and study designs. Moreover, studies evaluating these relationships in Indian populations are scarce. Given that atherogenic indices can be easily derived from routine lipid profiles and represent inexpensive, readily available biomarkers, their potential role in identifying patients with higher inflammatory burden warrants further investigation. Therefore, the present cross-sectional study aimed to evaluate the association between lipid-derived atherogenic indices and disease activity in patients with rheumatoid arthritis and to explore their potential utility as accessible markers of inflammatory burden. By examining the relationship between lipid-derived atherogenic indices and established measures of disease activity, this study seeks to contribute to the growing body of evidence regarding the interplay between dysregulated lipid metabolism and inflammatory activity in RA.

The Atherogenic Index of Plasma (AIP), calculated as log10(triglycerides/HDL-C), is a composite lipid-derived marker that reflects the balance between atherogenic and anti-atherogenic lipoproteins. Beyond its association with small dense LDL particles, AIP has been proposed as an integrated biomarker reflecting interactions among lipid metabolism, chronic inflammation, and oxidative stress, making it particularly relevant in inflammatory disorders such as rheumatoid arthritis [10,11].

The primary objective of this study was to evaluate the association between atherogenic indices and disease activity in patients with rheumatoid arthritis. The secondary objectives were to compare lipid profile parameters and atherogenic indices between patients with rheumatoid arthritis and control participants without rheumatoid arthritis and to examine the relationship between atherogenic indices and inflammatory markers.

## Materials and methods

Study design and setting

This cross-sectional observational study was conducted at the Department of Medicine, Pacific Medical College and Hospital, Udaipur, Rajasthan, India, between July 2023 and December 2024. The study aimed to evaluate the association between atherogenic indices and disease activity in patients with rheumatoid arthritis (RA). In addition, age- and sex-matched healthy controls were recruited to compare lipid profile parameters and atherogenic indices between patients with RA and individuals without inflammatory rheumatic disease. The study protocol was approved by the Institutional Ethics Committee (IEC), Pacific Medical College and Hospital (approval no. PMU/PMCH/IEC/GEN/2023/131; approval date: 27 June 2023). Written informed consent was obtained from all participants before enrollment. The study was conducted in accordance with the ethical principles of the Declaration of Helsinki.

Study population

A total of 150 participants were included in the study, comprising 100 patients with rheumatoid arthritis and 50 healthy controls. Consecutive patients attending the Medicine Outpatient Department at Pacific Hospital during the study period were screened for eligibility. Patients aged 18 years or older who fulfilled the 2010 American College of Rheumatology/European League Against Rheumatism (ACR/EULAR) classification criteria for rheumatoid arthritis were included in the study [1]. Control participants were recruited from hospital staff, attendants, and community volunteers and were frequency matched to cases by age and sex. Controls had no history of rheumatoid arthritis or other inflammatory rheumatic diseases. However, the presence of common non-inflammatory comorbidities such as hypertension or diabetes mellitus was not an exclusion criterion and was documented during clinical assessment. Participants with diabetes mellitus, chronic kidney disease, chronic liver disease, hypothyroidism, established cardiovascular disease, active infection, malignancy, pregnancy, or other autoimmune rheumatic disorders were excluded. Individuals receiving lipid-lowering medications, including statins or fibrates, were also excluded to minimize potential confounding effects on lipid parameters.

Sample size calculation

The sample size was calculated based on the primary objective of assessing the correlation between atherogenic indices and disease activity, as measured by Disease Activity Score in 28 joints with C-reactive protein (DAS28-CRP), in patients with rheumatoid arthritis. Fisher's z-transformation method for correlation studies was used, assuming an anticipated correlation coefficient (r) of 0.30, a two-sided alpha error of 0.05, and a study power of 80%. The minimum required sample size was estimated to be 85 patients. After accounting for a 15% allowance for incomplete data and potential exclusions, the final sample size was increased to 100 patients with rheumatoid arthritis. Additionally, 50 age- and sex-matched control participants without rheumatoid arthritis or other inflammatory rheumatic diseases were recruited

Clinical assessment

Demographic and clinical information was collected using a structured case record form. Data recorded included age, sex, disease duration, smoking status, medication history, and relevant comorbidities. Anthropometric measurements were obtained using standardized techniques. Height and weight were measured with participants wearing light clothing and no footwear. Body mass index (BMI) was calculated as weight in kilograms divided by height in meters squared (kg/m²). Waist circumference was measured using a non-stretchable measuring tape at the midpoint between the lower margin of the last palpable rib and the iliac crest at the end of normal expiration, with participants standing upright. Hypertension was defined as a previous physician diagnosis, current use of antihypertensive medication, or blood pressure ≥140/90 mmHg. Diabetes mellitus was defined as a previous physician diagnosis, current use of antidiabetic medication, fasting plasma glucose ≥126 mg/dL, or glycated hemoglobin (HbA1c) ≥6.5%, according to standard diagnostic criteria.

Clinical assessment and blood sample collection for inflammatory markers and lipid profile were performed during the same study visit, ensuring that DAS28-CRP assessment and calculation of atherogenic indices were based on contemporaneous clinical and laboratory data. Disease activity was assessed using the DAS28-CRP, which incorporates the 28-joint tender joint count, 28-joint swollen joint count, patient global assessment (100-mm visual analogue scale), and serum C-reactive protein (CRP) concentration. DAS28-CRP scores were calculated using the validated DAS28-CRP methodology described by Wells et al. [12], which was derived from the original DAS28 developed by Prevoo et al. [13]. Disease activity was interpreted using the DAS28-CRP cut-off values proposed by Inoue et al. [14], classifying patients as having remission (<2.6), low (2.6-3.2), moderate (>3.2-5.1), and high (>5.1) disease activity. The DAS28 is a published, validated clinical assessment instrument and does not require permission or licensing for non-commercial academic research. The ACR/EULAR classification criteria and DAS28-CRP are widely used clinical research instruments and do not require permission or licensing fees for academic and non-commercial research use.

Laboratory investigations

Following an overnight fast of 8-12 hours, venous blood samples were collected from all participants. Routine laboratory investigations included erythrocyte sedimentation rate (ESR), C-reactive protein (CRP), rheumatoid factor (RF), and anti-citrullinated protein antibody (ACPA) status in patients with RA. Venous blood samples were collected after an overnight fast. Serum total cholesterol, triglycerides, HDL-C, LDL-C, and other biochemical parameters were analyzed using an automated biochemistry analyzer (Beckman Coulter AU680) following the manufacturer's instructions and standard laboratory protocols. Serum C-reactive protein (CRP) was measured using an immunoturbidimetric assay, while erythrocyte sedimentation rate (ESR) was determined by the Westergren method.

Calculation of atherogenic indices

Atherogenic indices were calculated from fasting lipid parameters using established formulas described in previous studies [4-6]. Castelli Risk Index-I (CRI-I) was calculated as: CRI-I = Total Cholesterol/HDL-C. Castelli Risk Index-II (CRI-II) was calculated as: CRI-II = LDL-C/HDL-C. Atherogenic Index of Plasma (AIP) was calculated as: AIP = log10 (Triglycerides/HDL-C). For AIP calculation, triglyceride and HDL-C concentrations were converted to mmol/L before analysis [4]. Atherogenic Coefficient (AC) was calculated as: AC = (Total Cholesterol − HDL-C)/HDL-C. Non-HDL Cholesterol was calculated as: Non-HDL-C = Total Cholesterol − HDL-C. These indices are based on published formulas and do not require permission for academic or research use [4-6].

Data management and quality control

Data were entered into a secure electronic database and independently verified for accuracy. Records were reviewed for completeness and consistency before analysis. Continuous variables were assessed for normality using the Shapiro-Wilk test and graphical methods, including histograms and Q-Q plots.

Statistical analysis

Statistical analyses were performed using IBM SPSS Statistics, version 26 (IBM Corp., Armonk, NY, USA). Continuous variables were expressed as mean ± standard deviation (SD) for normally distributed data and median with interquartile range (IQR) for non-normally distributed data. Categorical variables were summarized as frequencies and percentages. Comparisons between RA patients and healthy controls were performed using the independent samples t-test for normally distributed variables and the Mann-Whitney U test for non-normally distributed variables. The Chi-square test or Fisher's exact test was used for categorical variables.

Differences in lipid parameters and atherogenic indices across disease activity categories were evaluated using one-way analysis of variance (ANOVA) with post-hoc Tukey correction for normally distributed variables or the Kruskal-Wallis test for non-parametric variables. Effect sizes for ANOVA were reported using partial eta squared (ηp²). Associations between atherogenic indices and disease activity measures were assessed using Pearson's correlation coefficient or Spearman's rank correlation coefficient, as appropriate. Correlations were evaluated between atherogenic indices and DAS28-CRP, ESR, and CRP. Medication use, including methotrexate, corticosteroids, and biologic therapies, was evaluated as a potential confounder in exploratory analyses.

To identify factors independently associated with high disease activity (DAS28-CRP >5.1), binary logistic regression analysis was performed using high disease activity as the dependent variable. Variables with p-values <0.10 in univariate analysis and considered clinically relevant were evaluated for multicollinearity using variance inflation factors (VIFs). Variables with VIF values >5 were considered to exhibit substantial multicollinearity. Because significant collinearity was observed among lipid-derived indices (CRI-I, CRI-II, atherogenic coefficient, and non-HDL-C), only the Atherogenic Index of Plasma (AIP), which demonstrated the strongest univariate association with disease activity, was retained in the final multivariable model. Binary logistic regression was then performed using BMI, ESR, and AIP as candidate predictors. Results are presented as regression coefficients (β), standard errors (SE), odds ratios (ORs) with 95% confidence intervals (CIs), and corresponding p-values. Model fit was assessed using the Hosmer-Lemeshow goodness-of-fit test. All statistical tests were two-tailed, and a p-value <0.05 was considered statistically significant.

## Results

Demographic and anthropometric characteristics of study participants

**Figure 1:**
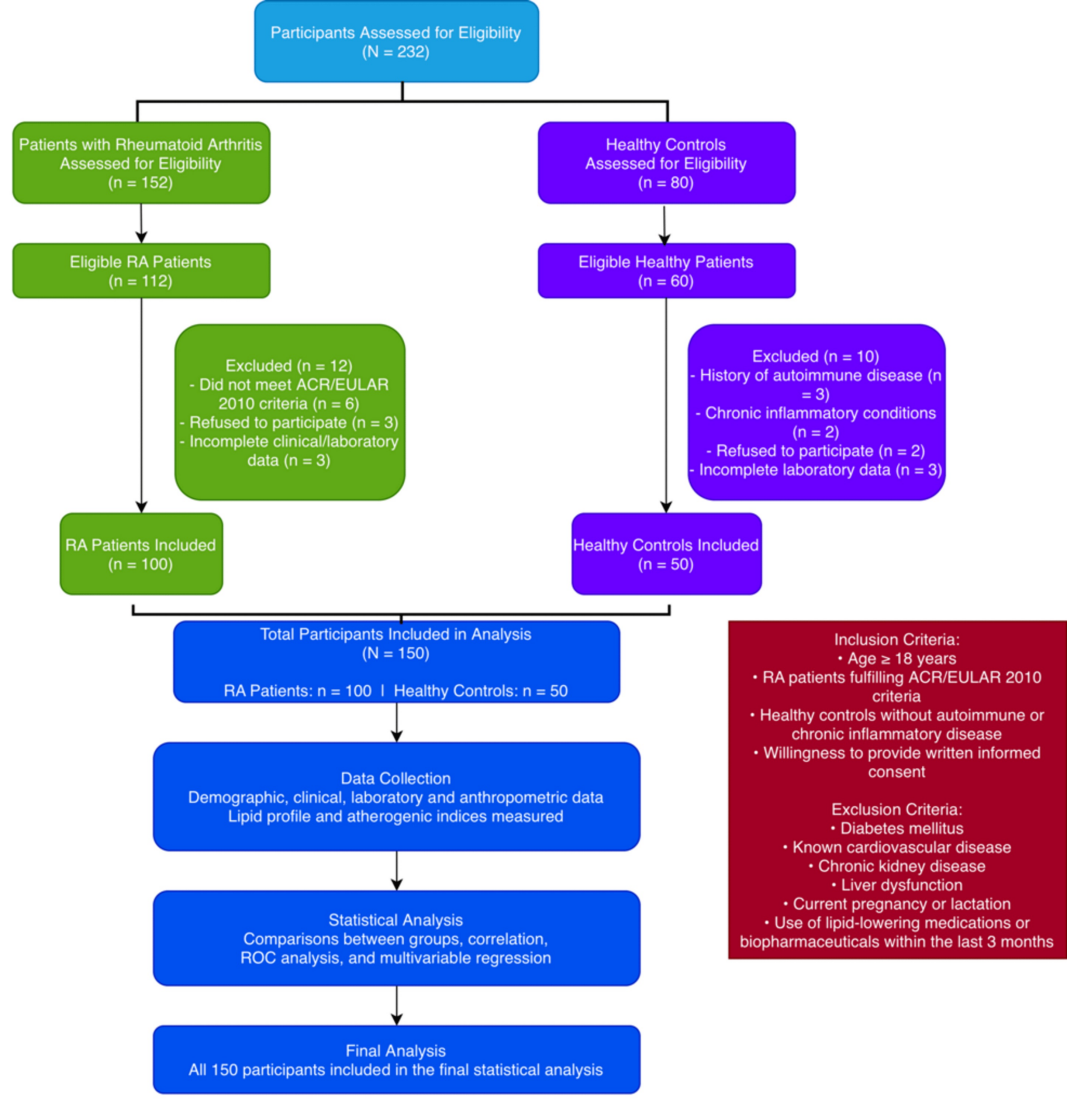
Flow diagram of participant recruitment and study inclusion Flow diagram illustrating participant screening, eligibility assessment, exclusions, and final inclusion of patients with rheumatoid arthritis (RA) and healthy controls. A total of 232 individuals were screened for eligibility, of whom 150 participants (100 RA patients and 50 healthy controls) met the study criteria and were included in the final analysis. Demographic, clinical, anthropometric, laboratory, lipid profile, and disease activity data were collected from all participants. Statistical analyses included group comparisons, correlation analyses, analysis of variance (ANOVA), and multivariable logistic regression. RA = Rheumatoid arthritis; ACR = American College of Rheumatology; EULAR = European League Against Rheumatism; ROC = Receiver operating characteristic.

A total of 150 participants were included in the study, comprising 100 patients with rheumatoid arthritis (RA) and 50 control participants. The demographic and anthropometric characteristics of the study population are summarized in Table 1. The two groups were comparable with respect to age (50.4 ± 10.8 vs. 48.7 ± 11.9 years, p = 0.39) and sex distribution (female: 80.0% vs. 80.0%, p = 1.00). The control group comprised individuals without rheumatoid arthritis or other inflammatory rheumatic diseases, although some participants had common non-inflammatory comorbidities such as hypertension or diabetes mellitus. Patients with RA exhibited significantly higher body mass index (BMI) (26.1 ± 4.1 vs. 24.4 ± 3.5 kg/m², p = 0.014), greater waist circumference (91.8 ± 9.5 vs. 85.8 ± 8.7 cm, p < 0.001), and a higher prevalence of hypertension (28.0% vs. 12.0%, p = 0.026) than the control group (Table 1). The participant recruitment process, eligibility assessment, and final study population are summarized in Figure 1.

**Table 1:** Demographic and anthropometric characteristics of study participants Data are presented as Mean ± SD for continuous variables and n (%) for categorical variables. Comparisons between rheumatoid arthritis (RA) patients and healthy controls were performed using an independent-samples t-test for continuous variables and a Chi-square (χ²) test for categorical variables. Statistical significance was defined as p < 0.05.

Parameter	RA Patients (n=100)	Controls (n=50)	Test Statistic	Effect Size	p-value
Age (years), Mean ± SD	50.4 ± 10.8	48.7 ± 11.9	t(148)=0.86	d = 0.15	0.39
Female sex, n (%)	80 (80.0)	40 (80.0)	χ²(1)=0.00	φ = 0.00	1.00
BMI (kg/m²), Mean ± SD	26.1 ± 4.1	24.4 ± 3.5	t(148)=2.49	d = 0.44	0.014
Waist circumference (cm), Mean ± SD	91.8 ± 9.5	85.8 ± 8.7	t(148)=3.66	d = 0.66	<0.001
Hypertension, n (%)	28 (28.0)	6 (12.0)	χ²(1)=4.94	φ = 0.18	0.026
Diabetes mellitus, n (%)	17 (17.0)	4 (8.0)	χ²(1)=2.24	φ = 0.12	0.13
Current smokers, n (%)	15 (15.0)	4 (8.0)	χ²(1)=1.44	φ = 0.10	0.23

Clinical and disease characteristics of patients with rheumatoid arthritis

The clinical characteristics of patients with RA are presented in Table 2. The mean disease duration was 7.4 ± 4.8 years, and the mean DAS28-CRP score was 4.56 ± 1.21. Rheumatoid factor and anti-citrullinated protein antibody positivity were observed in 76.0% and 72.0% of patients, respectively. Based on DAS28-CRP, 12.0% of patients were in remission, 18.0% had low disease activity, 45.0% had moderate disease activity, and 25.0% had high disease activity.

**Table 2:** Clinical and disease characteristics of patients with rheumatoid arthritis Data are presented as Mean ± SD, Median (IQR), or n (%), as appropriate. This table is descriptive; therefore, no between-group statistical comparisons were performed. N = 100 rheumatoid arthritis patients. DAS28-CRP = Disease Activity Score in 28 joints with C-reactive protein; ESR = Erythrocyte sedimentation rate; CRP = C-reactive protein; RF = Rheumatoid factor; ACPA = Anti-citrullinated protein antibodies.

Parameter	RA Patients (n=100)
Disease duration (years), Mean ± SD	7.4 ± 4.8
DAS28-CRP, Mean ± SD	4.56 ± 1.21
ESR (mm/hr), Mean ± SD	41.8 ± 17.6
CRP (mg/L), Median (IQR)	13.8 (6.9–24.3)
RF positivity, n (%)	76 (76.0)
ACPA positivity, n (%)	72 (72.0)
Methotrexate use, n (%)	79 (79.0)
Steroid use, n (%)	61 (61.0)
Biologic therapy use, n (%)	10 (10.0)
Remission (DAS28-CRP <2.6), n (%)	12 (12.0)
Low disease activity (2.6–3.2), n (%)	18 (18.0)
Moderate disease activity (3.2–5.1), n (%)	45 (45.0)
High disease activity (>5.1), n (%)	25 (25.0)

Lipid profile and atherogenic indices comparison

Comparison of lipid parameters revealed a significantly more atherogenic lipid profile among patients with RA than controls (Table 3). Triglycerides, LDL-C, VLDL-C, and non-HDL-C were significantly elevated in the RA group, whereas HDL-C levels were significantly lower (all p ≤ 0.003). Similarly, all evaluated atherogenic indices were significantly higher in patients with RA, including CRI-I (4.48 ± 1.11 vs. 3.54 ± 0.82), CRI-II (2.82 ± 0.78 vs. 2.02 ± 0.61), AIP (0.39 ± 0.18 vs. 0.14 ± 0.12), and AC (3.48 ± 1.08 vs. 2.55 ± 0.79) (all p < 0.001). No significant difference was observed in total cholesterol levels between groups (p = 0.15). 

**Table 3:** Lipid profile and atherogenic indices in rheumatoid arthritis patients and healthy controls Data are presented as Mean ± Standard Deviation (SD). Comparisons between patients with rheumatoid arthritis (RA) and healthy controls were performed using the independent samples Student's t-test. Mean differences with 95% confidence intervals (CIs), t-statistics (t(148)), and corresponding p-values are reported. Statistical significance was defined as p < 0.05. HDL-C = High-density lipoprotein cholesterol; LDL-C = Low-density lipoprotein cholesterol; VLDL-C = Very-low-density lipoprotein cholesterol; CRI-I = Castelli Risk Index-I; CRI-II = Castelli Risk Index-II; AIP = Atherogenic Index of Plasma; AC = Atherogenic Coefficient.

Parameter	RA (n=100)	Controls (n=50)	Mean Difference (95% CI)	Test Statistic	Cohen's d	p-value
Total Cholesterol (mg/dL)	186.8 ± 34.2	179.4 ± 29.7	7.4 (−2.8 to 17.6)	t(148)=1.44	0.23	0.15
Triglycerides (mg/dL)	168.4 ± 48.6	127.6 ± 35.8	40.8 (25.3–56.3)	t(148)=5.12	0.95	<0.001
HDL-C (mg/dL)	41.8 ± 8.5	50.6 ± 8.7	−8.8 (−11.7 to −5.9)	t(148)=−5.91	1.02	<0.001
LDL-C (mg/dL)	116.2 ± 28.3	102.7 ± 24.1	13.5 (4.8–22.2)	t(148)=3.04	0.51	0.003
VLDL-C (mg/dL)	33.7 ± 9.7	25.5 ± 7.2	8.2 (5.0–11.4)	t(148)=5.01	0.93	<0.001
Non-HDL-C (mg/dL)	145.0 ± 31.1	128.8 ± 27.2	16.2 (6.3–26.1)	t(148)=3.25	0.55	0.001
TC/HDL-C Ratio (CRI-I)	4.48 ± 1.11	3.54 ± 0.82	0.94 (0.58–1.30)	t(148)=5.16	0.96	<0.001
LDL/HDL-C Ratio (CRI-II)	2.82 ± 0.78	2.02 ± 0.61	0.80 (0.54–1.06)	t(148)=6.04	1.12	<0.001
AIP	0.39 ± 0.18	0.14 ± 0.12	0.25 (0.19–0.31)	t(148)=8.12	1.48	<0.001
Atherogenic Coefficient	3.48 ± 1.08	2.55 ± 0.79	0.93 (0.58–1.28)	t(148)=5.23	0.97	<0.001

**Figure 2:**
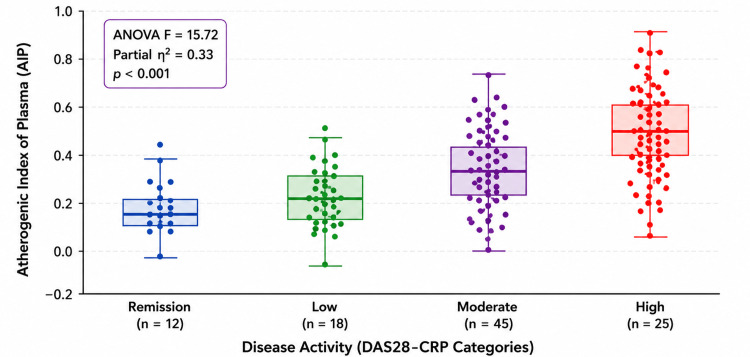
Distribution of atherogenic index of plasma (AIP) across rheumatoid arthritis disease activity categories Box-and-whisker plots showing the distribution of the AIP across rheumatoid arthritis disease activity categories defined according to DAS28-CRP: remission (n = 12), low disease activity (n = 18), moderate disease activity (n = 45), and high disease activity (n = 25). The horizontal line within each box represents the median, the box indicates the interquartile range (IQR), and whiskers represent 1.5 × IQR. Individual data points are displayed to illustrate data distribution. Comparisons among groups were performed using one-way analysis of variance (ANOVA) with Tukey's post-hoc test. AIP increased progressively with increasing disease activity (ANOVA F = 15.72, partial η² = 0.33, p < 0.001). Statistical significance was defined as p < 0.05. AIP = Atherogenic Index of Plasma; DAS28-CRP = Disease Activity Score in 28 Joints using C-reactive Protein; ANOVA = Analysis of variance.

A progressive increase in atherogenic indices was observed across RA disease activity categories (Table 4, Figure 2). Patients with high disease activity demonstrated the highest values for CRI-I, CRI-II, AIP, AC, and non-HDL-C. Among the lipid-derived markers, AIP exhibited the strongest association with disease activity category (F = 15.72, partial η² = 0.33, p < 0.001), indicating a large effect size. Significant differences were also observed for CRI-II (F = 11.46, partial η² = 0.27, p < 0.001), CRI-I (F = 9.82, partial η² = 0.24, p < 0.001), and AC (F = 10.85, partial η² = 0.25, p < 0.001). Inflammatory markers increased in parallel with disease activity, with ESR and CRP demonstrating large effect sizes (partial η² = 0.46 and 0.52, respectively).

**Table 4:** Comparison of atherogenic indices across rheumatoid arthritis disease activity categories Data are presented as Mean ± SD. Comparisons among disease activity categories were performed using one-way analysis of variance (ANOVA). F statistics, partial eta squared (ηp²) effect sizes, and corresponding p-values are reported. Post-hoc pairwise comparisons were performed using Tukey's test where applicable. Statistical significance was defined as p < 0.05. Interpretation of effect size (partial η²): Small = 0.01, Moderate = 0.06, Large = 0.14. CRI-I = Castelli Risk Index-I; CRI-II = Castelli Risk Index-II; AIP = Atherogenic Index of Plasma; AC = Atherogenic Coefficient; ESR = Erythrocyte sedimentation rate; CRP = C-reactive protein.

Parameter	Remission (n=12) Mean ± SD	Low Activity (n=18) Mean ± SD	Moderate Activity (n=45) Mean ± SD	High Activity (n=25) Mean ± SD	F(3,96)	Partial η²	p-value
TC/HDL-C Ratio (CRI-I)	3.65 ± 0.62	4.02 ± 0.71	4.51 ± 0.85	5.08 ± 1.02	9.82	0.24	<0.001
LDL/HDL-C Ratio (CRI-II)	2.08 ± 0.49	2.34 ± 0.55	2.79 ± 0.68	3.26 ± 0.78	11.46	0.27	<0.001
Atherogenic Index of Plasma (AIP)	0.18 ± 0.11	0.24 ± 0.13	0.37 ± 0.16	0.52 ± 0.19	15.72	0.33	<0.001
Atherogenic Coefficient (AC)	2.65 ± 0.63	3.05 ± 0.71	3.52 ± 0.82	4.10 ± 0.95	10.85	0.25	<0.001
Non-HDL-C (mg/dL)	126 ± 24	133 ± 26	145 ± 29	157 ± 33	6.34	0.17	0.001
ESR (mm/hr)	23 ± 9	31 ± 11	44 ± 15	60 ± 18	27.51	0.46	<0.001
CRP (mg/L)	6.1 ± 3.0	9.4 ± 4.6	15.2 ± 6.8	27.8 ± 10.2	34.82	0.52	<0.001

**Figure 3:**
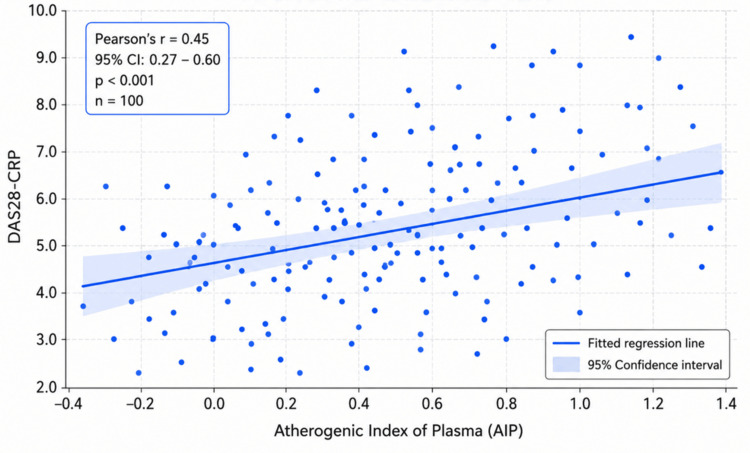
Correlation between Atherogenic Index of Plasma (AIP) and DAS28-CRP in patients with rheumatoid arthritis Scatter plot illustrating the relationship between the AIP and DAS28-CRP among patients with rheumatoid arthritis (n = 100). Each point represents an individual participant. The solid line represents the fitted linear regression line, and the shaded area indicates the 95% confidence interval. Pearson's correlation analysis demonstrated a significant positive association between AIP and DAS28-CRP (r = 0.45, 95% CI: 0.27–0.60; p < 0.001). Statistical significance was defined as p < 0.05. AIP = Atherogenic Index of Plasma; DAS28-CRP = Disease Activity Score in 28 Joints using C-reactive protein; CI = Confidence interval.

Correlation analyses demonstrated significant positive associations between atherogenic indices and disease activity measures (Table 5). AIP showed the strongest correlation with DAS28-CRP (r = 0.45, p < 0.001), followed by CRI-II (r = 0.38, p < 0.001), AC (r = 0.36, p < 0.001), and CRI-I (r = 0.34, p < 0.001). Similar positive correlations were observed with ESR and CRP. Figure 3 illustrates the significant positive relationship between AIP and DAS28-CRP.

**Table 5:** Correlation between atherogenic indices and disease activity parameters in patients with rheumatoid arthritis Data are presented as Pearson's correlation coefficients (r). Correlations between atherogenic indices and disease activity parameters were evaluated using Pearson's correlation analysis. Corresponding p-values are reported for each correlation coefficient. Statistical significance was defined as p < 0.05. DAS28-CRP = Disease Activity Score in 28 Joints using C-reactive Protein; ESR = Erythrocyte sedimentation rate; CRP = C-reactive protein; CRI-I = Castelli Risk Index-I; CRI-II = Castelli Risk Index-II; AIP = Atherogenic Index of Plasma; Non-HDL-C = Non-high-density lipoprotein cholesterol.

Atherogenic Index	DAS28-CRP (r)	p (DAS28-CRP)	ESR (r)	p (ESR)	CRP (r)	p (CRP)
CRI-I	0.31	0.002	0.28	0.005	0.25	0.012
CRI-II	0.37	<0.001	0.33	0.001	0.30	0.003
AIP	0.45	<0.001	0.39	<0.001	0.36	<0.001
Atherogenic Coefficient	0.35	<0.001	0.31	0.002	0.28	0.005
Non-HDL-C (mg/dL)	0.24	0.016	0.21	0.036	0.18	0.074

Multicollinearity assessment revealed substantial collinearity among CRI-I, CRI-II, atherogenic coefficient, and non-HDL-C (all VIF >5). Therefore, only AIP was retained in the final multivariable logistic regression model (Table 6).

**Table 6:** Multivariable logistic regression analysis for factors independently associated with high disease activity (DAS28-CRP >5.1) Binary logistic regression analysis was performed to identify factors independently associated with high disease activity (DAS28-CRP >5.1). Variables included in the final model were selected based on clinical relevance, univariate significance, and assessment of multicollinearity. Results are presented as β regression coefficients, standard errors (SE), Wald χ² statistics, odds ratios (ORs), and 95% confidence intervals (CIs). Model calibration was assessed using the Hosmer–Lemeshow goodness-of-fit test. Statistical significance was defined as p < 0.05. BMI = Body Mass Index; ESR = Erythrocyte sedimentation rate; AIP = Atherogenic Index of Plasma; OR = Odds ratio; CI = Confidence interval; SE = Standard error.

Variable	β Coefficient	Standard Error	Wald χ²	OR	95% CI	p-value
BMI (kg/m²)	0.077	0.039	3.88	1.08	1.00–1.17	0.049
ESR (mm/hr)	0.030	0.011	8.12	1.03	1.01–1.05	0.004
AIP	1.138	0.399	8.14	3.12	1.42–6.85	0.004
Constant	-5.284	1.842	8.23	—	—	0.004

Multicollinearity assessment revealed substantial collinearity among CRI-I, CRI-II, AC, and non-HDL-C (all variance inflation factor values >5). Consequently, only AIP was retained in the final multivariable logistic regression model. As shown in Table 6, BMI (OR = 1.08, 95% CI: 1.00-1.17, p = 0.049), ESR (OR = 1.03, 95% CI: 1.01-1.05, p = 0.004), and AIP (OR = 3.12, 95% CI: 1.42-6.85, p = 0.004) were independently associated with high disease activity. 

## Discussion

The present study evaluated the relationship between lipid-derived atherogenic indices and disease activity in patients with rheumatoid arthritis (RA) and demonstrated several important findings. Patients with RA exhibited significantly more adverse lipid profiles and higher atherogenic indices than healthy controls. Furthermore, atherogenic indices increased progressively across disease activity categories and showed significant positive correlations with established measures of inflammation, including DAS28-CRP, ESR, and CRP. Among the evaluated markers, the Atherogenic Index of Plasma (AIP) demonstrated the most consistent association with disease activity across multiple analytical approaches, including group comparisons, correlation analyses, disease activity stratification, and multivariable regression. These findings support the concept that dysregulated lipid metabolism and inflammatory disease activity are closely interconnected in RA.

The increased cardiometabolic burden observed among patients with RA is consistent with current evidence demonstrating that chronic systemic inflammation contributes to accelerated atherosclerosis and increased cardiovascular morbidity and mortality [1]. Persistent inflammatory activity promotes endothelial dysfunction, oxidative stress, insulin resistance, and alterations in lipoprotein metabolism, thereby increasing cardiovascular risk. The high prevalence of metabolic abnormalities reported in RA populations worldwide further supports this association. In a recent systematic review and meta-analysis, Cai et al. estimated that nearly one-third of patients with RA met the criteria for metabolic syndrome, emphasizing the substantial cardiometabolic burden associated with the disease [2].

In the present study, patients with RA had significantly higher triglyceride, LDL-C, VLDL-C, and non-HDL-C levels, together with significantly lower HDL-C levels than healthy controls. These findings are consistent with those reported by Bhattacharya et al., who observed elevated atherogenic indices and significant associations between metabolic abnormalities and disease activity in Indian patients with RA [3]. Similarly, Dessie demonstrated significant relationships between atherogenic indices, inflammatory markers, and cardiovascular risk factors, suggesting that lipid-derived indices may reflect both inflammatory and metabolic disturbances in RA [4]. Our findings also align with those of Sreekumar et al., who highlighted the high prevalence of atherothrombotic risk factors among patients with RA and emphasized the importance of comprehensive cardiovascular risk assessment in routine clinical practice [5].

A notable observation in the present study was the progressive increase in atherogenic indices across disease activity categories. Disease activity was assessed using the validated Disease Activity Score in 28 joints with C-reactive protein (DAS28-CRP), and all participants fulfilled the 2010 American College of Rheumatology/European League Against Rheumatism (ACR/EULAR) classification criteria for rheumatoid arthritis, ensuring a well-defined study population [10]. The DAS28 scoring system developed by Prevoo et al. remains one of the most widely accepted composite measures for evaluating RA disease activity [13], while the DAS28-CRP thresholds used in the present study have been shown to closely correspond to conventional DAS28-ESR disease activity categories. Patients with high disease activity exhibited the highest values across all evaluated atherogenic indices, whereas those in remission showed the lowest values. AIP showed the largest effect size across disease activity categories (partial η² = 0.33) and a moderate positive correlation with DAS28-CRP (r = 0.45), suggesting a meaningful relationship between lipid abnormalities and inflammatory burden. These findings are consistent with those reported by Grzechnik and Targowska-Stepniak, who found that patients with metabolic syndrome exhibited significantly higher disease activity and inflammatory marker levels than those without metabolic abnormalities [6]. However, not all studies have reported similar findings. Tekaya and Rouached observed a high prevalence of metabolic syndrome among patients with RA but did not identify a significant association with disease activity measures [7]. Differences in study populations, treatment patterns, ethnicity, and definitions of metabolic abnormalities may partly explain these divergent findings. 

The present findings may also be interpreted in the context of the "lipid paradox" observed in rheumatoid arthritis, whereby patients with active inflammatory disease may exhibit increased cardiovascular risk despite apparently normal or reduced concentrations of conventional lipid parameters. Chronic systemic inflammation alters lipoprotein composition and function, particularly by increasing triglyceride-rich lipoproteins and impairing the anti-inflammatory properties of HDL particles. Inflammatory cytokines promote metabolic lipid reprogramming, oxidative stress, and persistent inflammatory signaling, while excess lipid accumulation within synovial tissues may contribute to local lipotoxicity and progressive joint damage. Consequently, composite lipid-derived indices such as AIP may better reflect the combined effects of dysregulated lipid metabolism and inflammatory burden than isolated lipid parameters alone.

The prominence of AIP observed in the present study is biologically plausible. AIP integrates information from both triglyceride and HDL-C concentrations and has been shown to correlate with the presence of small dense LDL particles and increased atherogenicity [14,15]. Similarly, the Castelli Risk Indices (CRI-I and CRI-II), which were also significantly associated with disease activity in the present study, are based on the ratio of total cholesterol or LDL-C to HDL-C and have long been recognized as indicators of adverse lipid metabolism [16]. Collectively, these lipid-derived indices provide complementary information regarding the balance between atherogenic and anti-atherogenic lipoproteins. Supporting this concept, Ge et al. reported a significant association between elevated AIP and arthritis prevalence in a large population-based analysis of NHANES data, suggesting broader links between dysregulated lipid metabolism and inflammatory musculoskeletal disorders [8]. Similarly, emerging evidence indicates that atherogenic indices may reflect both inflammatory activity and cardiometabolic risk in rheumatoid arthritis, supporting their potential clinical utility in routine patient assessment [9].

Multivariable logistic regression demonstrated that AIP remained independently associated with high disease activity after adjustment for BMI and ESR. Although these findings should not be interpreted as evidence of causality due to the cross-sectional design, they suggest that AIP may provide clinically relevant information complementary to established inflammatory markers. Because AIP can be calculated easily from routine lipid profiles without additional cost or specialized investigations, it may represent a practical adjunctive marker for assessing inflammatory disease activity and dysregulated lipid metabolism, particularly in resource-constrained healthcare settings. Disease duration was evaluated as a clinically relevant variable because longer disease duration may contribute to cumulative inflammatory burden and cardiometabolic risk. Although patients with longer-standing disease tended to exhibit higher disease activity, disease duration was not independently associated with high disease activity in multivariable analyses. This finding suggests that current inflammatory status may have a greater influence on atherogenic alterations than disease chronicity alone.

Corticosteroid therapy may also have influenced the observed lipid profile and atherogenic indices. Although glucocorticoids effectively suppress systemic inflammation, their metabolic effects are dose- and duration-dependent. Low-dose corticosteroid therapy may improve lipid profiles indirectly through better disease control, whereas prolonged or higher-dose therapy has been associated with dyslipidemia, insulin resistance, and increased cardiovascular risk. Because detailed cumulative corticosteroid exposure was not available for all participants, the independent effect of steroid therapy on atherogenic indices could not be evaluated in the present study. Future studies incorporating corticosteroid dose and duration are warranted to better define their influence on lipid-derived cardiovascular risk markers in rheumatoid arthritis.

Several limitations of this study should be acknowledged. The cross-sectional design precludes assessment of temporal or causal relationships between atherogenic indices and disease activity. The study was conducted at a single center, which may limit generalizability to other populations. The control group was recruited from hospital staff, patient attendants, and community volunteers rather than through population-based sampling; therefore, selection bias cannot be completely excluded. Additionally, the potential influence of disease-modifying antirheumatic drugs, corticosteroids, and biologic therapies on lipid parameters and inflammatory activity could not be fully accounted for. In particular, detailed information on cumulative corticosteroid dose and duration was not available for all participants, precluding evaluation of their independent effects on atherogenic indices. Disease duration was evaluated but did not demonstrate an independent association with high disease activity in multivariable analyses. Nevertheless, the inclusion of a well-characterized cohort, comprehensive assessment of multiple atherogenic indices, incorporation of effect-size estimates, and multicollinearity-adjusted regression analysis strengthen the validity of the findings.

Future longitudinal and multicenter studies are needed to determine whether changes in atherogenic indices parallel treatment response, predict long-term cardiovascular outcomes, and improve risk stratification beyond conventional inflammatory markers. Further research should also explore clinically meaningful cut-off values and evaluate whether incorporation of atherogenic indices into routine disease monitoring strategies can enhance the comprehensive management of patients with rheumatoid arthritis. Future studies should also investigate whether rheumatoid factor and anti-cyclic citrullinated peptide antibody seropositivity influence atherogenic indices and cardiovascular risk profiles in patients with rheumatoid arthritis.

## Conclusions

This cross-sectional study demonstrated that patients with rheumatoid arthritis exhibited significantly more adverse lipid profiles and higher atherogenic indices than control participants without rheumatoid arthritis. Atherogenic indices increased progressively with increasing disease activity and showed significant associations with established inflammatory markers, including DAS28-CRP, ESR, and CRP. Among the evaluated lipid-derived markers, the Atherogenic Index of Plasma (AIP) demonstrated the most consistent relationship with disease activity across correlation analyses, disease activity stratification, and multivariable regression. These findings suggest that lipid-derived atherogenic indices, particularly AIP, may serve as practical adjunctive markers of inflammatory disease activity in rheumatoid arthritis.

The present study adds to the growing body of evidence linking dysregulated lipid metabolism with rheumatoid arthritis disease activity and provides novel data from an Indian cohort. Because these indices can be derived easily from routine lipid profiles without additional cost or specialized investigations, they may complement conventional clinical and laboratory assessments in routine practice. Future longitudinal and multicenter studies are warranted to determine whether changes in atherogenic indices parallel treatment response and improve disease monitoring and risk stratification in patients with rheumatoid arthritis.
